# Benefits of a Disease Management Program for Sickle Cell Disease in Germany 2011–2019: The Increased Use of Hydroxyurea Correlates with a Reduced Frequency of Acute Chest Syndrome

**DOI:** 10.3390/jcm10194543

**Published:** 2021-09-30

**Authors:** Joachim B. Kunz, Andreas Schlotmann, Andrea Daubenbüchel, Stephan Lobitz, Andrea Jarisch, Regine Grosse, Holger Cario, Lena Oevermann, Dani Hakimeh, Laura Tagliaferri, Andreas E. Kulozik

**Affiliations:** 1 Hopp-Children’s Cancer Center (KiTZ), Department of Pediatric Oncology, Hematology and Immunology, Heidelberg, University of Heidelberg, Im Neuenheimer Feld 430, 69120 Heidelberg, Germany; andrea.daubenbuechel@med.uni-heidelberg.de (A.D.); laura.tagliaferri@med.uni-heidelberg.de (L.T.); Andreas.Kulozik@med.uni-heidelberg.de (A.E.K.); 2 GPOH Konsortium Sichelzellkrankheit, Chausseestraße 128/129, 10115 Berlin, Germany; stephan.lobitz@gk.de (S.L.); andrea.jarisch@kgu.de (A.J.); regine.grosse@gmx.de (R.G.); holger.cario@uniklinik-ulm.de (H.C.); lena.oevermann@charite.de (L.O.); dani.hakimeh@charite.de (D.H.); 3 Wissenschaftliches Institut der AOK, Integrierte Daten und Analysen, Rosenthaler Str. 31, 10178 Berlin, Germany; andreas.schlotmann@wido.bv.aok.de; 4 Pädiatrische Hämatologie und Onkologie, Gemeinschaftsklinikum Mittelrhein Kemperhof, Koblenzer Straße 115-155, 56073 Koblenz, Germany; 5 Schwerpunkt Stammzelltransplantation und Immunologie, Zentrum für Kinder- und Jugendmedizin, Klinikum der Johann-Wolfgang-Goethe-Universität, Theodor-Stern-Kai 7, 60590 Frankfurt am Main, Germany; 6 Zentrum für Geburtshilfe, Kinder- und Jugendmedizin, Klinik und Poliklinik für Pädiatrische Hämatologie und Onkologie, Universitätsklinikum Hamburg-Eppendorf, Martinistr. 52, 20246 Hamburg, Germany; 7 Pädiatrische Hämatologie und Onkologie, Klinik für Kinder- und Jugendmedizin, Universitätsklinikum Ulm, Eythstrasse 24, 89075 Ulm, Germany; 8 Campus Virchow-Klinikum, Klinik für Pädiatrie m.S. Onkologie/Hämatologie/KMT, Charité—Universitätsmedizin Berlin, Augustenburger Platz 1, 13353 Berlin, Germany

**Keywords:** Sickle Cell Disease, hydroxyurea, epidemiology, health insurance data

## Abstract

Sickle Cell Disease (SCD) is the most common monogenic disorder globally but qualifies as a rare disease in Germany. In 2012, the German Society for Paediatric Oncology and Haematology (GPOH) mandated a consortium of five university hospitals to develop a disease management program for patients with SCD. Besides other activities, this consortium issued treatment guidelines for SCD that strongly favour the use of hydroxyurea and propagated these guidelines in physician and patient education events. In order to quantify the effect of these recommendations, we made use of claims data that were collected by the research institute (WIdO) of the major German insurance company, the *Allgemeine Ortskrankenkasse* (AOK), and of publicly accessible data collected by the Federal Statistical Office (*Statistisches Bundesamt, Destatis*). While the number of patients with SCD in Germany increased from approximately 2200 in 2011 to approximately 3200 in 2019, important components of the recently issued treatment guidelines have been largely implemented. Specifically, the use of hydroxyurea has more than doubled, resulting in a proportion of approximately 44% of all patients with SCD being treated with hydroxyurea in 2019. In strong negative correlation with the use of hydroxyurea, the frequency of acute chest syndromes decreased. Similarly, the proportion of patients who required analgesics and hospitals admissions declined. In sum, these data demonstrate an association between the dissemination of treatment guidelines and changes in clinical practice. The close temporal relationship between the increased use of hydroxyurea and the reduction in the incidence of acute chest syndrome in a representative population-based analysis implies that these changes in clinical practice contributed to an improvement in key measures of disease activity.

## 1. Introduction

**Figure 1:**
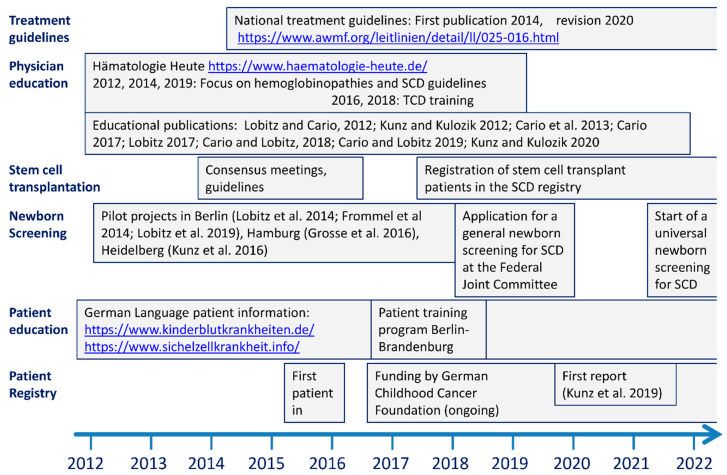
Disease management activities by the German SCD consortium. Physician education primarily included national guidelines [12] on the treatment of SCD that were disseminated by annual meetings and by publications in German language (see Supplementary References). Stem cell transplant physicians were enrolled in the consensus finding for the treatment guideline. Several pilot projects [6,7,8,9,10] prepared for a general new-born screening and led to a formal application at the Federal Joint Committee, the political body that recently decided the implementation of such a screening for 10/2021. Patient education was provided online and with the help of a structured training program at the Kindernachsorgeklinik Berlin Brandenburg, an institution specialized in rehabilitation of chronically ill children that closed by the end of 2019. Results of the national patient registry were reported elsewhere [5]. All websites that are mentioned in the Figure were accessed on 9 July 2021.

Sickle Cell Disease (SCD) is a devastating multi-organ disorder that is recessively inherited predominantly in populations with endemic malaria [1]. As a consequence of their colonial history, some European countries have already faced the challenges of SCD for decades and have established comprehensive care programs that rely on the combination of new born screening with centralizing treatment in dedicated centres [2,3]. With the surge of immigration from high prevalence regions, mainly sub-Saharan Africa and the middle east, during recent years, the number of patients with SCD in Germany has substantially increased [4,5], resulting in a birth prevalence of up to 1:2500 [6,7,8,9,10,11]. In an effort to optimize the management of SCD, the German Society of Paediatric Oncology and Haematology (GPOH) mandated a consortium of national experts with the implementation of a structured disease management program. This program (Figure 1) included the issuing of national treatment guidelines, educational activities for physicians and patients, pilot projects in new born screening for SCD [6,7,8,9,10] and the setup of a national registry for patients with SCD [5]. The treatment guidelines [12] are comparable to those in other countries [13,14], but more strongly recommend the use of hydroxyurea in patients with symptomatic SCD and include the use of allogeneic stem cell transplantation for those patients with an HLA-compatible sibling donor as standard treatment.

Currently, 753 patients are enrolled in a national registry for patients with SCD and most of these are being treated at referral centres by paediatric haematologists [5]. However, adults and patients who are taken care of by a family physician or a general practitioner are underrepresented because only few centres are dedicated to the care of adults with SCD and because hospital outpatient clinics are the main contributors to the registry [5]. In order to gain a representative overview on the epidemiology of SCD in Germany and the treatment that is administered, we have analysed the database of the research institute of the AOK (*Allgemeine Ortskrankenkasse),* the health insurance covering approximately one third of the total population in Germany and approximately 50% of the patients with SCD (see below). This database comprehensively collects data that are required for reimbursement, including diagnoses, procedures and prescribed medications of patients who are insured by the AOK.

Analysing this database, we aimed at providing core epidemiological data of SCD in Germany. In addition, comorbidities and claimed treatments have been quantified by using diagnoses documented with SCD and medications prescribed for patients with SCD. The burden of disease could thus be estimated based on the frequency of complications, the rate of annual hospitalizations and the number of prescribed doses of certain drugs. We hypothesize that changes in common clinical practice are reflected in changes of the burden of disease.

The data presented here demonstrate the benefit of a structured care program and serve as the basis for further improvements in the care of patients with SCD in Germany.

## 2. Materials and Methods

Study Design: The source population of this study were all persons insured with the AOK (*Allgemeine Ortskrankenkasse)*. The AOK, a system of eleven regional health care funds, insures approximately 26 million people and is Germany’s largest statutory health insurance fund. Children and adolescents with a migration background are overrepresented among AOK insures [15], as are adults with lower educational level and poor health status [16]. The database population consisted of anonymized data sets of all patients that were covered by the AOK health insurance for at least one full calendar year between 2011 and 2019.

Codes for diagnoses (based on the ICD10 classification) and procedures (based on OPS, German procedure classification) are entered into the AOK’s claims database by healthcare providers treating the patient and form the basis for remuneration by the health insurance. Prescribed medications are documented by public pharmacies using a modified ATC classification (Anatomical Therapeutic Chemical).

**Figure 2:**
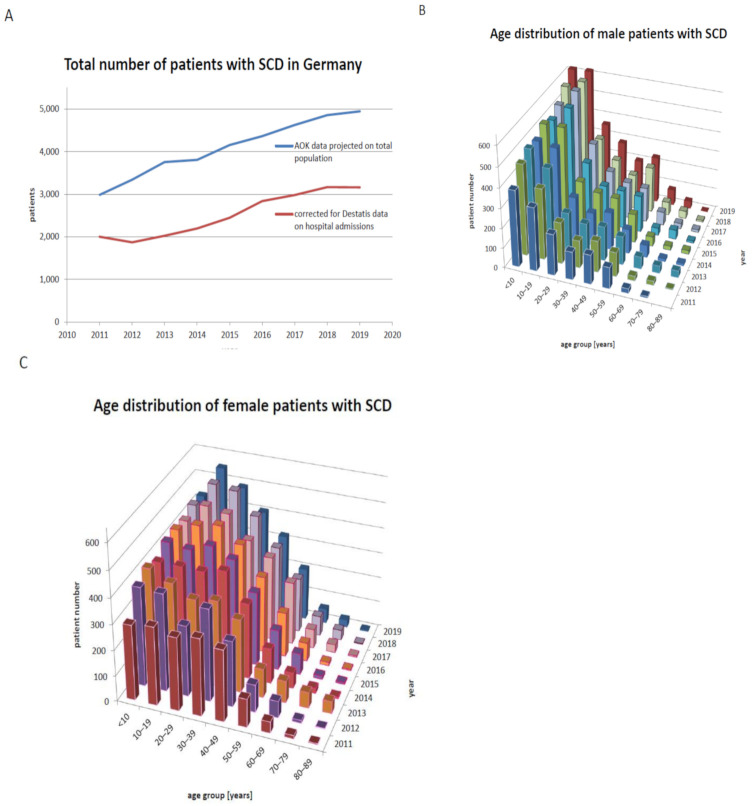
Number, sex and age of patients with SCD in Germany. (**A**) Estimated total number of patients with SCD in Germany based on AOK data and corrected by the Destatis data on hospital admissions, accounting for the suspected overrepresentation of migrants among those covered by AOK (see Supplementary Table S1 for correcting factors); (**B**) Distribution of age in male and (**C**) female patients with SCD 2011–2019.

For the estimate of the total number of patients with SCD (Figure 2), we chose three different definitions. First, a patient was defined as affected by SCD if the ICD10 codes D57.0, D57.1 or D57.2 had been documented, either by a haematologist or a paediatric haematologist or by a hospital outpatient clinic, or as a principal diagnosis leading to hospital admission (“diagnosis by experts”). Second, patients with the diagnosis documented by at least two independent physicians (no matter which specialization, including hospital diagnoses) were included (“two independent diagnoses”). Third, we combined all patients that fulfilled either of these definitions. Patients whose diagnosis by a non-haematologist was not confirmed by a second physician were excluded. This third definition was the basis for all subsequent analyses. The patient cohort with SCD as described above was characterized with respect to age, sex, hospitalizations and number of days spent in hospital per year, coexisting morbidities (pneumonia/acute chest syndrome J12-J18, stroke I60-I64, sepsis A39-A41, chronic kidney failure N18, pulmonary hypertension I27), number and age of fatalities.

Pharmacologic treatment was identified by the ATC codes of the prescribed medication: iron chelation (Deferoxamin V03AC01, Deferipron V03AC02, Deferasirox V03AC03), hydroxyurea (L01XX05), anti-inflammatory and anti-rheumatic products (M01), analgesics (N02) and opioids (N02A). A patient was considered as being prescribed a medication if at least one prescription of the respective ATC group in one calendar year was recorded.

Treatment with red blood cell transfusion for SCD was identified by the OPS codes 8–800.c (red blood cell transfusion, any number) or 8-801 (exchange transfusion).

Statistics: All numbers given refer to the total population in Germany, calculated based on the proportion of patients covered by the AOK insurance and corrected with respect to age and sex distributions.

In order to correct for a possible over- or underrepresentation of patients with SCD among AOK insurees in comparison to the general population, we made use of the numbers of hospital admissions with principal diagnosis D57.0 or D57.1 that were reported to the Federal Statistical Office (*Statistisches Bundesamt*, *Destatis*) and are publicly available [4,17]. We assumed that the number of hospital admissions was directly proportional to the number of patients with SCD. Thus, the ratio of admissions reported to Destatis over the admissions calculated based on the AOK data was defined as a “correction factor”. This correction factor varied between 2011 and 2019 from 0.54 to 0.67 (Supplementary Table S1), indicating that patients with SCD were overrepresented among AOK insurees. We multiplied the estimate for the total number of patients based on the AOK data with this correction factor in order to arrive at the final estimate for the total number of patients with SCD in Germany.

**Figure 3:**
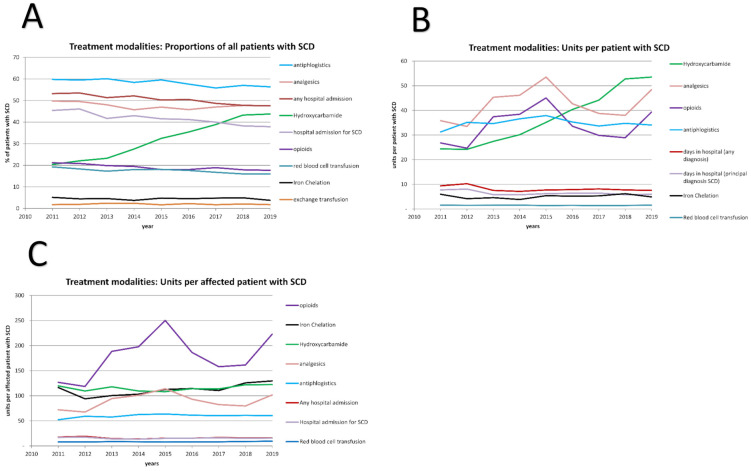
Treatment modalities administered in SCD patients. (**A**) The proportion of all patients with SCD for whom at least one hospital admission, one hospital admission with a diagnosis D57.0/1/2, red blood cell transfusions (OPS 8-800.c), iron chelation (ATC V03AC), or exchange transfusion (OPS 8-801), prescription of hydroxyurea (ATC L01XX05), anti-inflammatory and antirheumatic drug (M01), analgesics (N02) or opioids (N02A) per year was documented. (**B**) The different treatment modalities are quantified per patient with SCD as defined daily doses (DDD) for hydroxyurea, analgesics (M01, N02, N02A) and antichelating agents; for red blood cell transfusion, the number of red blood cell units transfused during an inpatient treatment and for hospital admissions, days in hospital were counted. The sum of these measures for all patients with SCD was divided by the number of patients with SCD. (**C**) As in (**B**), but the denominator was the number of patients with SCD that had at least one prescription of the treatment of interest. In order to account for the changing distribution of age and sex, patient numbers were normalized to the German population in 2019.

**Figure 4:**
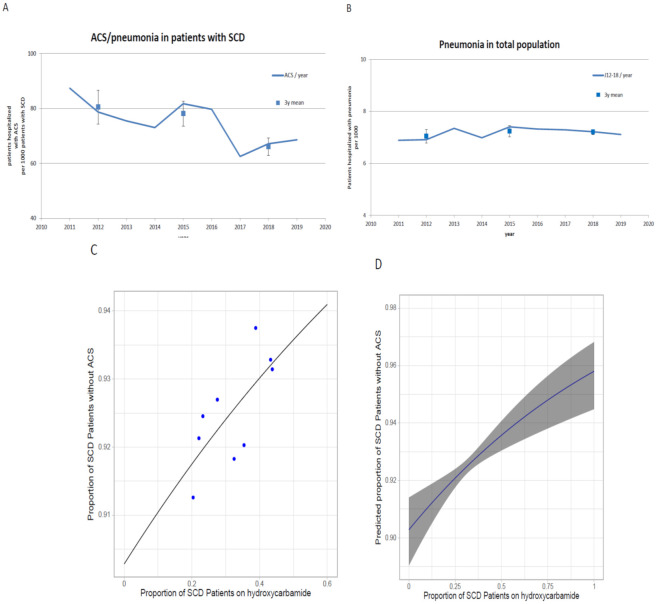
Acute chest syndrome/pneumonia in patients with SCD and in the general population. (**A**) Proportion of patients with SCD admitted with the diagnosis “pneumonia” (ICD10 J12–18) and (**B**) proportion of the total population admitted with the diagnosis “pneumonia” (J12–18). In order to account for the changing distribution of age and sex, patients’ numbers were normalized to the German population in 2019. Square: 3 years mean, error bars: standard deviation. (**C**,**D**): Visualization of a univariate logistic regression model evaluating the association between hydroxyurea treatment and ACS in patients with SCD. (**C**) Actual data (each dot represents one year) and crude regression line. (**D**) Predicted model with proportions of patients without ACS and 95% CI in dependence of the proportion of patients on hydroxyurea. Model estimates: y = (e^β^_0_^+xβ^_1_)/(1 + e^β^_0_^+xβ^_1_) with β_0_ = 2.229 (SE 0.069) and β_1_ = 0.009 (SE 0.002), x = proportion of patients on hydroxyurea.

In order to correct for the effect of changes in the distribution of sex and age over time on the frequency of complications of SCD, the usage of treatments and the frequencies of complications of SCD (Figure 3 and Figure 4) were standardized to the total population in Germany 2019.

A univariate logistic regression model was built to measure the relationship between hydroxycarbamide treatment and ACS in SCD patients. This analysis was performed using the statistical analysis software R version 4.1.0. Probabilities were estimated with the glm function. The variables included in the model were the proportion of SCD patients treated with hydroxycarbamide and the proportion of patients with occurrence of ACS. The latter was transformed into a binary variable. Actual values and model estimates including 95% confidence interval were plotted using package ggplot2.

## 3. Results

### 3.1. Estimation of the Number of Patients with SCD

In order to reliably estimate the number of patients with SCD and to eliminate “false positives” who are wrongly diagnosed as having SCD, we made use of three different definitions (see Methods Section 2). The number of patients who fulfilled any of the three definitions increased in parallel between 2011 and 2019 (Figure 2A, Supplementary Figure S1), mirroring the increase in hospital admissions with the principal diagnosis SCD in the same period [4]. The definition “diagnosis by experts” captured a higher proportion of children and adolescents below the age of 20 (57.1% of all patients) as compared to the definition “two independent diagnoses” (42.5%). The proportions of patients who were prescribed hydroxyurea, received red blood cell transfusion or were admitted to a hospital for the principal diagnosis of SCD in 2018 were similar for these two definitions (52.0 vs. 45.2%, 21.7 vs. 17.5%, 51.6 vs. 43.4%). We hypothesize that the difference in patient numbers fulfilling these definitions reflect age-related differences in the care of patients with SCD—that is, children and adolescents are more likely to be treated by a specialist, whereas adults are preferentially treated by general practitioners. While we cannot exclude that either definition erroneously includes some patients not truly affected by SCD, the consistently high proportion of patients treated with hydroxyurea and red blood cell transfusions prompted us to combine these two definitions for all further analyses (Figure 2A).

However, we have to be aware of the relatively high proportion of recent immigrants who are covered by the AOK insurance in comparison to the other statutory health insurances. To correct for this bias, we made use of the number of hospital admissions with principal diagnoses SCD that have been reported by the Federal Statistical Office for 2017 [17] and assumed that the number of hospital admissions is directly proportional to the patient number (see Methods Section 2 and Supplementary Table S1). With this correction, we arrived at a final estimate of 2005 for the total number of patients with SCD in Germany in 2011 and 3160 in 2019, corresponding to an increase by approximately 58% (Figure 2A).

During 2011 and 2019, not only the numbers of patients with SCD increased, but also the distribution of age and sex changed (Figure 2B,C). In absolute numbers, the age group below 30 years showed the strongest increase, consistent with the age distribution of immigrants arriving in Germany during this period [4]. In comparison to male patients, there is a striking surplus of female patients aged 20 to 49 years, possibly reflecting a higher life expectancy or, alternatively, a higher health care usage in female patients, resulting in a more complete detection by the selection criteria used. Consequently, the median age of female patients with SCD (28 years in 2011, 25 in 2019) was higher than the median age of male patients with SCD (18 in 2011, 19 in 2019).

### 3.2. Treatment of SCD Changed from 2011 to 2019

The most important treatment options for SCD include the induction of foetal haemoglobin with hydroxyurea, red blood cell transfusions and symptomatic treatment with analgesics. In order to assess changes in common practice, we analysed the proportion of patients with SCD who received these treatment modalities at least once per calendar year. From 2011 to 2019, the proportion of patients who were prescribed hydroxyurea approximately doubled from 20.4 to 43.8% (Figure 3A), resulting in a parallel increase in the number of daily doses prescribed per patient with SCD (Figure 3B). The daily dose per patient who was prescribed hydroxyurea remained constant (Figure 3C), corresponding to approximately 552 mg/d. The proportion of patients with SCD undergoing exchange transfusions and iron chelation remained constant at 2 and 5%, respectively.

### 3.3. Changes in Comorbidities and in the Burden of Disease

We hypothesized that the increased use of hydroxyurea resulted in a measurable decrease in the burden of SCD and aimed at quantifying changes in the frequency of relevant complications of SCD. In order to correlate the burden of disease with the use of hydroxyurea, we compared the period before the widespread use of hydroxyurea, from 2011 to 2013 (mean proportion of patients on hydroxyurea 21.9%, range 20.4% to 23.2%) with the period from 2017 to 2019 (mean proportion of patients on hydroxyurea 42.0%, range 38.9% to 43.8%). Between 2011–2013 and 2017–2019, the proportion of patients with SCD admitted to hospital for any reason decreased from 52.7% (range 51.3%–53.5%) to 48% (range 47.5%–48.7%, *p* Student’s *t*-test = 0.009, Figure 3A). The same trend was observed for hospital admissions with the principal diagnosis SCD (44.4% to 38.7%, *p* = 0.035, Figure 3A). Further, the days spent in hospital decreased between 2011–13 and 2017–19 from 9.04 days to 7.79 days per patient and year (14%, *p* = 0.27, Figure 3B). In the total population, the proportion that was admitted to hospital decreased by less than 3%, the days spent in hospital by 10% (*p* = 0.021 and 0.003, respectively; Supplementary Figure S2).

The analysis of complications of SCD was limited by several factors. First, stroke (I60-I69, <5 per 1000), sepsis (A40-41; <20 per 1000 patients with SCD per year) and renal failure (N18; <20 per 1000) affected too few patients to identify possible trends with time. Second, there is no separate ICD code for the most frequent complication of SCD, acute painful vasoocclusive crises. We therefore focused on acute chest syndrome, the most frequent complication of SCD that is coded with a separate ICD10 code, J12-18 (“pneumonia”). Randomized controlled trials observed a reduction in acute chest syndromes with the increased use of hydroxyurea [18,19]. Compatible with previous reports [5,20], approximately 7% of patients with SCD were admitted with the diagnosis J12-18 in our data set (Figure 4A). This number exceeds that of patients with pneumonia in the general population (Figure 4B) approximately tenfold and decreased by 18% from 2011–13 to 2017–19 (*p* = 0.0002, Chi-Square). In parallel, the incidence of ACS/pneumonia in patients with SCD decreased from 98 to 82 per 1000 patient years ( Figure S3a). No such trend was observed for admissions for pneumonia in the total population (Figure 4B, Supplementary Figure S3b).

We evaluated the association between the usage of hydroxyurea and the frequency of ACS/pneumonia in patients with SCD using a univariate logistic regression model. The estimates of this model suggest a significant relationship between hydroxyurea treatment and ACS/pneumonia (*p* ≤ 0.001). With an increase in the proportion of patients treated with hydroxyurea by 10%, the proportion of patients with ACS/pneumonia decreases by 0.54% (Figure 4C,D).

**Figure 5:**
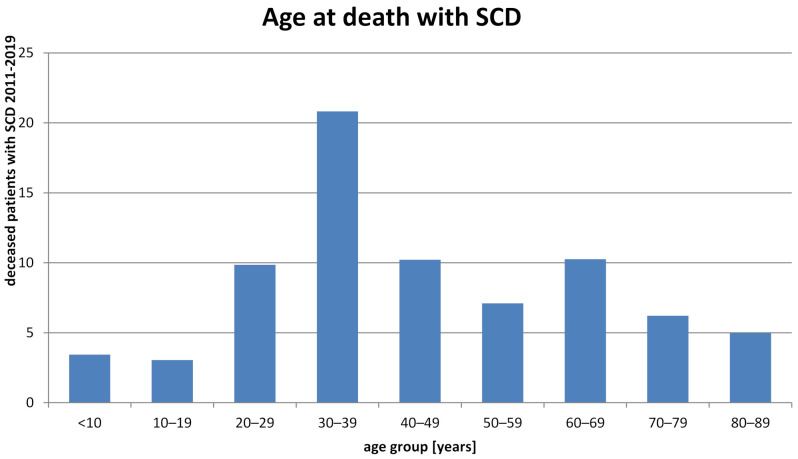
Age at death in patients with SCD. Age distribution of patients with SCD who died in the period 2011–2019.

As expected for a young cohort of patients with SCD, few patients died during the observation period. The group with the highest absolute number of fatalities is that of patients aged between 20 and 39 years, whereas children and adolescents show a lower risk of death (median age at death 37, Figure 5). However, because of the lack of a new-born screening program for SCD in Germany, it is unknown how many young patients may have died of SCD before the correct diagnosis has been made. The ratio of fatalities among male patients to those among female patients was 1.68 and higher than the ratio of male patients with SCD to female patients with SCD (2011: 0.82, 2019: 0.98, Figure 2B,C), indicating a higher mortality in male patients with SCD compared to female patients with SCD. The relatively high number of patients who were diagnosed to suffer from SCD and died at an age of >60 years implies that there may be a considerable number of erroneous diagnoses in this age group. As our analysis is based on ICD codes that do not specify the different genotypes of SCD, we can only speculate that patients dying at an old age with the diagnoses SCD may have suffered from Hb SC disease or HbS/β^+^thal disease.

## 4. Discussion

### 4.1. Epidemiology of SCD in Germany

Our estimate for the number of patients with SCD that is based on health insurance data, 3160 in 2019, is consistent with previous estimates. Based on the number of immigrants from countries with endemic SCD, the number of immigrants with SCD in Germany has been estimated to be 3216 in 2015 [4]. German citizens with SCD would add to this number. Data from the German SCD registry resulted in an estimate of at least 2000 patients with SCD [5]. The concordance between these different estimates and the large proportion of the population in Germany that is covered by the AOK database is reassuring. These data indicate that the number of patients with SCD is indeed increasing and has exceeded the number of 3000 in 2019.

With the strongest increase in the age groups between 10 and 29 years since 2011, most likely due to the immigration of predominantly young adults from regions with a high SCD prevalence, patients with SCD remain considerably younger than the general population. The overrepresentation of patients in the reproductive age compared to the general population explains why the birth prevalence of SCD (approximately 1:5000 [11]) is higher than the prevalence in the general population (approximately 1:25,000).

### 4.2. Changes in Treatment of SCD and Changes in the Burden of Disease

The use of hydroxyurea has considerably increased between 2013 and 2019. This remarkable change coincides with the time of the publication and dissemination of the current treatment guidelines [12], suggesting that physicians taking care of patients with a condition outside of their previous daily routine have eagerly implemented these guidelines, including the prescription of hydroxyurea. Taking into account the proportion of patients who do not generally have an indication for hydroxyurea, most importantly patients with HbSC disease and children below the age of two, approximately 50% of patients are prescribed hydroxyurea. This proportion is much higher than in most other healthcare systems [3,21,22,23]. Unfortunately, the data only refer to the prescribed and dispensed treatment and do not allow conclusions on patient adherence. Nevertheless, the trend towards a reduction in acute chest syndrome was stronger than expected. Randomized trials had demonstrated a reduction in the frequency of acute chest syndrome by approximately 50% in adults [19] and 70% in infants [24] with the use of hydroxyurea. Based on these results, increasing the proportion of patients treated with hydroxycarbamide by 20% is predicted to result in a reduction in the frequency of acute chest syndromes by approximately 10 to 14%, a number that is even exceeded by the effect observed in our population-based data set. The parallel strong trends for a reduction in acute chest syndrome, for hospital admissions and for the use of analgesics indicate that the increased prescription of hydroxyurea that followed the active dissemination of treatment guidelines had a positive clinical effect.

### 4.3. Limitations

Our study suffers from several limitations. The most important one is that these anonymized observational analyses are based on health data that have been collected for reimbursement purposes and are not validated as clinical trial data would be. By contrast, reimbursement relevant insurance data have been subjected to multiple levels of plausibility controls that are likely more rigorous than used in many conventional retrospective analyses. Nevertheless, we cannot exclude that some of the patients who were included were wrongly diagnosed with SCD. This may especially be relevant for the unexpectedly high number of patients diagnosed with SCD above the age of 70. If we assume that all diagnoses in this age group were made erroneously and that the same absolute number of misdiagnoses had been made in all other age groups, the estimate for the total number of patients with SCD would need to be corrected by approximately 7%. In contrast, patients with SCD may have been missed because of the absence of a general new-born screening for SCD. In addition, our stringent inclusion criteria excluded patients who have been taken care of exclusively by a non-haematologist physician, e.g., a family practitioner, thus possibly underestimating the number of SCD patients.

Several important aspects of SCD could not be addressed because of an insufficient documentation in the insurance database. For example, we could not analyse patient data according to genotype because the ICD classification does not reliably differentiate homozygous and compound heterozygous SCD. Similarly, the ICD code for patients who received stem cell transplantation (Z94.8) does not discriminate patients cured from those with recurrence of SCD due to autologous reconstitution. The former may even be missed because the ICD code D57 does not further apply. Although the number of patients with SCD who were treated with stem cell transplantation and registered with the German Registry for Stem Cell Transplantation (DRST) has increased during recent years, their total was limited to 192 in the period of 2011 to 2019 [25], indicating this potential confounder does not play a major role.

Because the AOK covers approximately a third of the German population and an estimated 50% of the patients with SCD, these “real world data” identify national trends that are not reflected by data from clinical trials or registries. Despite this very broad database, the patients covered by the AOK may not be representative for the total population Germany. One obvious bias is a higher proportion of SCD patients among AOK insurees in comparison to the general population [15]. While we accounted our estimate of the total number of patients with SCD for this bias, we do not know if this overrepresentation of recent immigrants skewed the frequencies of treatment modalities and even complications.

While the strength of these routinely collected health data is the representative coverage of a large proportion of all patients, we cannot conclude on the course of individual patients. In order to gain a comprehensive view that accounts for both the entirety of patients with SCD and for the individual courses of single patients, routinely collected health data need to be complemented by registry data. Results from a national registry that predominantly enrols paediatric patients taken care of by academic institutions showed that approximately 80% of patients were prescribed hydroxyurea, a proportion exceeding that of the general population twice [5]. These numbers indicate that the care for patients with SCD depends on the caregiver and may be improved by offering care from specialized centres. While these are well established for children and adolescents, more institutions providing specialized care for adults with hemoglobinopathies are needed to further reduce morbidity and mortality associated with SCD.

## 5. Conclusions

This real-world data analysis gives evidence that treatment habits can change in close temporal relationship with the publication of guidelines if these guidelines are actively disseminated and promoted among physicians and patients. With current treatment guidelines, we observe an increase in the use of hydroxyurea in patients with SCD that is linked to a decrease in the incidence of acute chest syndromes. The clinical benefits for the patients that are associated with these changes should be a strong motivation to foster national disease management programs, even for apparently rare disorders such as SCD in Germany.

## Data Availability

Data that is visualized in Figure 2, Figure 3, Figure 4 and Figure 5 is available with publication at request to the corresponding author.

## Supplementary Materials

The following are available online at https://www.mdpi.com/article/10.3390/jcm10194543/s1. Figure S1: Number of patients with SCD according to different working definitions. Figure S2: Treatment modalities in the total population. Figure S3: Frequency of acute chest syndrome in patients with SCD and of pneumonia in the general population. Table S1: Correction factors for the overrepresentation of patients with SCD among AOK insures. Supplementary references [1–8].
