# Supplementary material for: Benefits of a Disease Management Program for Sickle Cell Disease in Germany 2011–2019: The Increased Use of Hydroxyurea Correlates with a Reduced Frequency of Acute Chest Syndrome
Source: J Clin Med. 2021 Sep 30;10(19):4543. doi: 10.3390/jcm10194543 (PMC8509263; doi:10.3390/jcm10194543)
Supplement: Supplementary file 1 [file jcm-10-04543-s001.zip › jcm-1382644-Supplementary.pdf]

Benefits of a Disease Management Program for Sickle Cell Disease in Germany 2011 - 2019: The Increased Use of Hydroxyurea Correlates with a Reduced Frequency of Acute Chest Syndrome

Joachim B. Kunz, Andreas Schlotmann, Andrea Daubenbüchel, Stephan Lobitz, Andrea Jarisch, Regine Grosse, Holger Cario, Lena Oevermann, Dani Hakimeh, Laura Tagliaferri and Andreas E. Kulozik

Supplementary Material

Table S1: Correction factors for the overrepresentation of patients with SCD among AOK insurees. We made use of the numbers of hospital admissions with principal diagnosis D57.0 or D57.1 that were reported to the Federal Statistical Office (Statistisches Bundesamt, Destatis) and assumed that the number of hospital admissions was directly proportional to the number of patients with SCD. Thus, the ratio of admissions reported to Destatis over the admissions calculated based on the AOK data was defined as a "correction factor". In order to obtain an estimate for the total number of patients with SCD in Germany, the raw estimate based on the AOK data was multiplied with the respective correction factor.

Year	Correction factor
2011	0.672
2012	0.560
2013	0.539
2014	0.578
2015	0.590
2016	0.651
2017	0.645
2018	0.652
2019	0.639

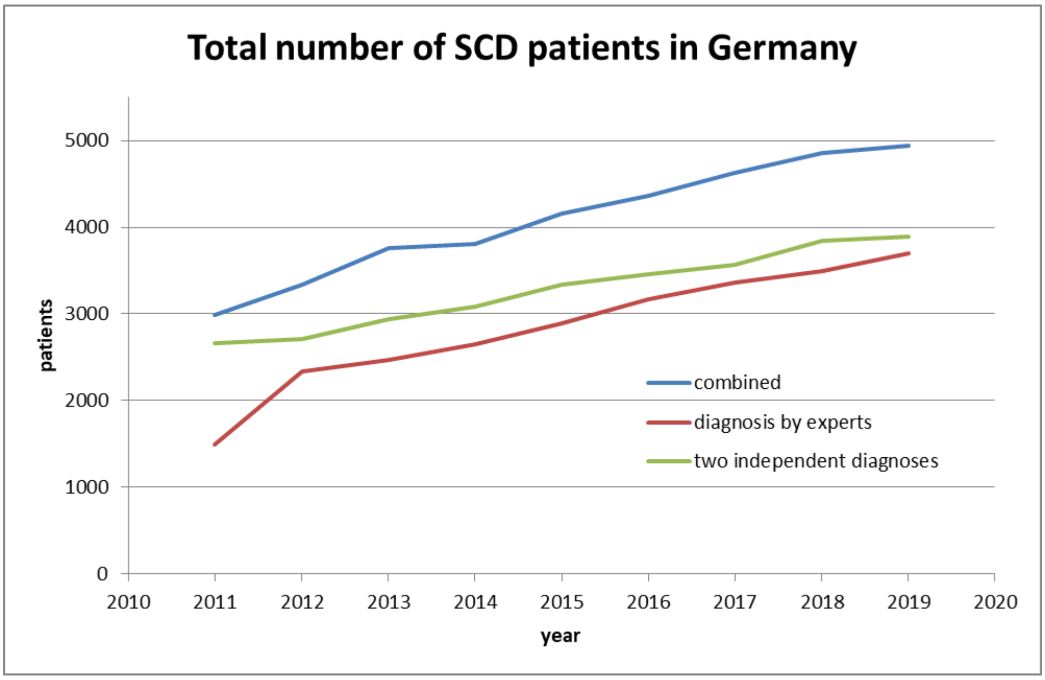

Figure S1: Number of patients with SCD according to different working definitions. For all patients defined as affected by SCD the ICD10 codes D57.0, D57.1 or D57.2 were documented. Carriers of the sickle cell trait (D57.3) were excluded. If the diagnosis was made by a hematologist or a pediatric hematologist or by a hospital outpatient clinic, or as a principal diagnosis leading to hospital admission, the patient fulfilled the definition "diagnosis by experts". Patients with the diagnosis documented by at least two independent physicians (no matter which specialization, including hospital diagnoses) were included in the definition "two independent diagnoses". Finally, patients that fulfilled either the definition "diagnosis by experts" or "two independent diagnoses" were combined ("combined"). Patients whose diagnosis by a non-hematologist was not confirmed by a second physician were excluded.

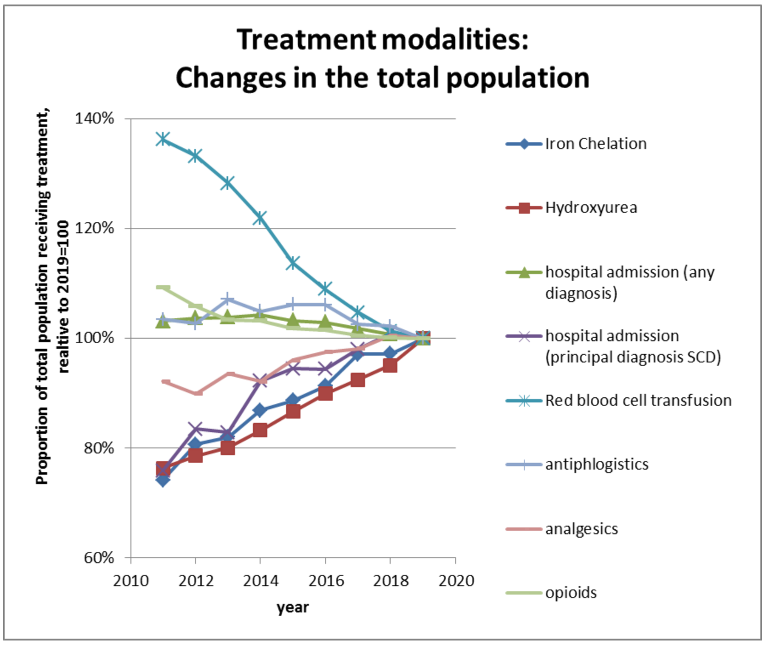
Treatment modalities:
Changes in the total population
Proportion of total population receiving treatment, realtive to 2019=100
140%
120%
100%
80%
60%
2010
2012
2014
2016
2018
2020
year
Iron Chelation
Hydroxyurea
hospital admission (any diagnosis)
hospital admission (principal diagnosis SCD)
Red blood cell transfusion
antiphlogistics
analgesics
opioids

(a)

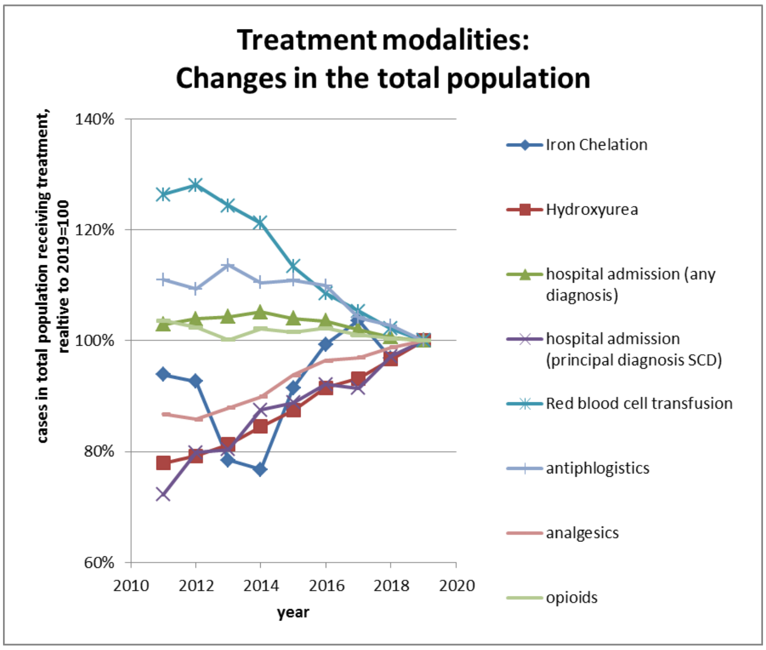
Treatment modalities:
Changes in the total population
cases in total population receiving treatment, realtive to 2019=100
140%
120%
100%
80%
60%
2010
2012
2014
2016
2018
2020
year
Iron Chelation
Hydroxyurea
hospital admission (any diagnosis)
hospital admission (principal diagnosis SCD)
Red blood cell transfusion
antiphlogistics
analgesics
opioids

(b)

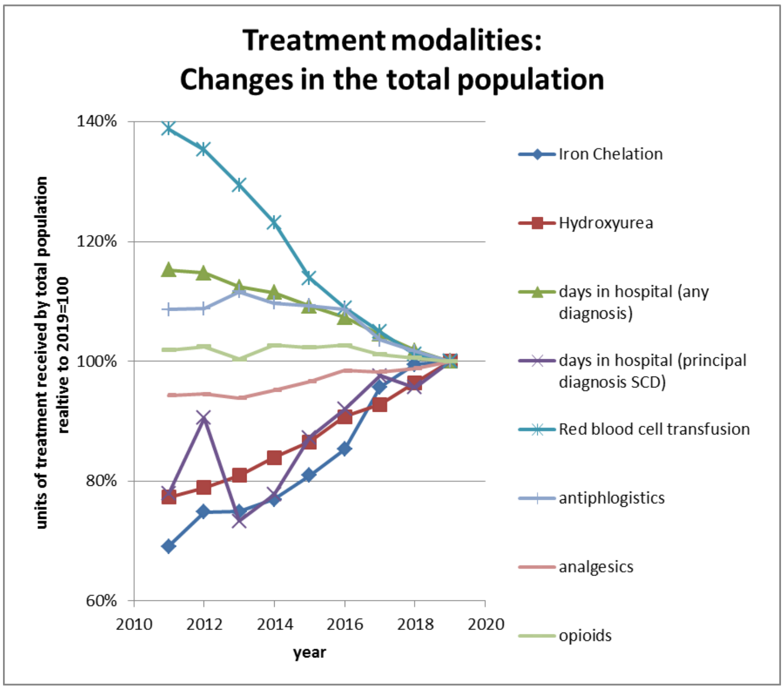

(c)

Figure S2: Treatment modalities in the total population. (**a**) Changes in the proportion of the population for whom at least one hospital admission, one hospital admission with a diagnosis D57.0/1/2, red blood cell transfusions (OPS 8-800.c), iron chelation (ATC V03AC), prescription of hydroxycarbamide (ATC L01XX05), anti-inflammatory and antirheumatic drugs (M01), analgesics (N02) or opioids (N02A) per year was documented. The proportion in 2019 was set to 100% (**b**) As in a, but cases, not distinct patients were counted. 2019 was set to 100%. "Cases" refers to prescriptions (for hydroxyurea, iron chelation, antiphlogistics, analgesics, opioids) or hospital admissions od procedures (red blood cell transfusions) (**c**) Quantitative changes in different treatment modalities as defined daily doses (DDD) for hydroxycarbamide, analgesics (M01, N02, N02A) and antichelating agents; for red blood cell transfusion: the number of red blood cell units transfused during an inpatient treatment and for hospital admissions days in hospital were counted. The quantity per person in 2019 was set to 100%.

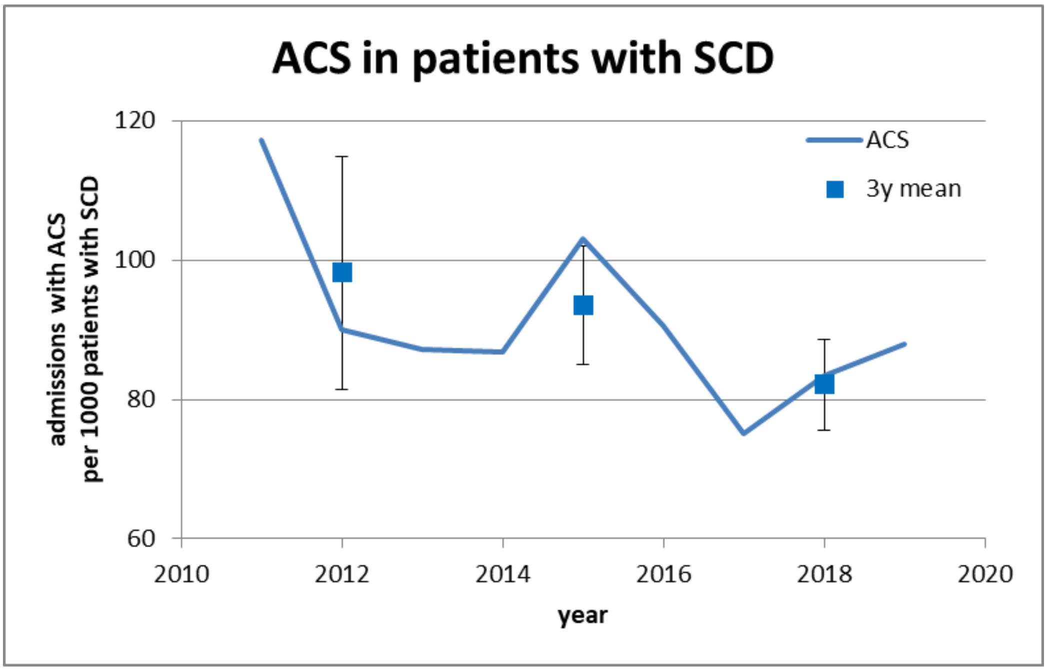

(a)

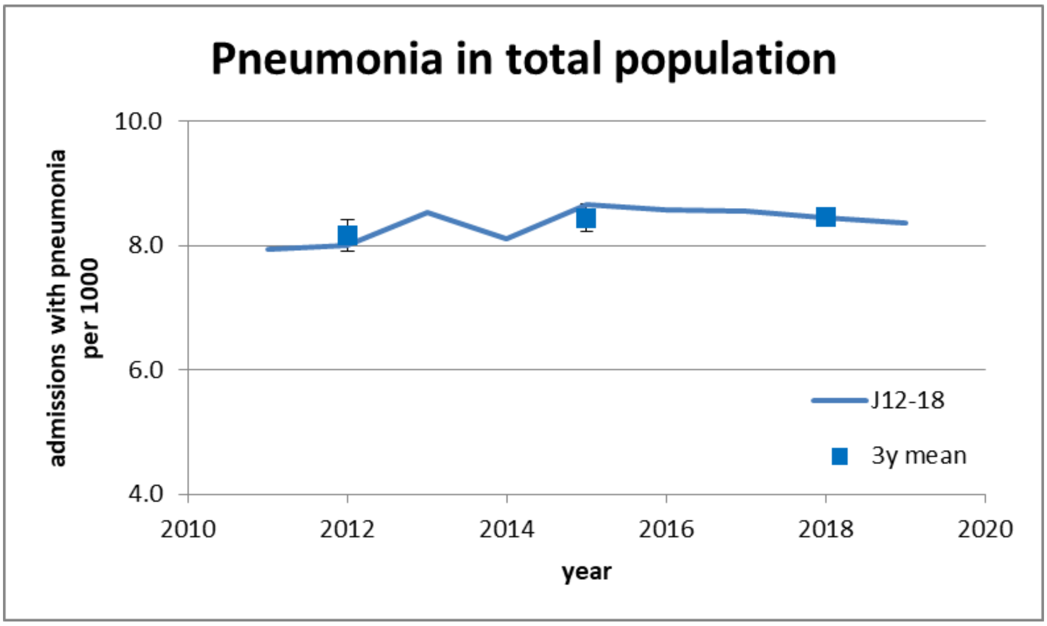

(b)

Figure S3. Frequency of acute chest syndrome in patients with SCD and of pneumonia in the general population. (**a**) Admissions per 1000 patients with SCD with the diagnosis "acute chest syndrome" (J12-18) and (**b**) admissions in the total population admitted with the diagnosis "pneumonia" (J12-18). In order to account for the changing distribution of age and sex, patients numbers were normalized to the German population in 2019. Red square: 3 years mean, error bars: standard deviation.

Supplementary References

1. Cario H, Lobitz S. Was ist neu in der Diagnostik und Therapie der Hämoglobinopathien? *DMW - Deutsche Medizinische Wochenschrift* 2019; **144**: 719-23.
2. Cario H, Lobitz S. Hämoglobinopathien als Herausforderung der Migrantenmedizin. *Monatsschrift Kinderheilkunde* 2018; (11).
3. Cario H, Weinstock C, Mayer B, Lobitz S. Grundlagen und Besonderheiten der Transfusionstherapie bei Hämoglobinopathien. *Transfusionsmedizin - Immunhämatologie · Hämotherapie · Transplantationsimmunologie · Zelltherapie* 2013; **3**(02): 92-110.
4. Lobitz S, Cario H. Sichelzellkrankheit. *Kinder- und Jugendmedizin* 2012; **31**(05): 314-21.
5. Cario H. „Migrantenanämien" oder „Migrationsanämie". *Kinder- und Jugendmedizin* 2017; **17**(02): 75-80.
6. Lobitz S. Perspektive: Neugeborenen -screening auf Sichelzellkrankheiten in Deutschland. *Kinder- und Jugendmedizin* 2017; **17**(02): 82-6.
7. Kunz J, Kulozik A. Aktualisierte Handlungsempfehlung nach der Leitlinie „Anämiediagnostik im Kindesalter". *Monatsschrift Kinderheilkunde* 2020; **168**(7): 644-6.
8. Kunz JB, Kulozik AE. Differenzialdiagnose der kindlichen Anämie. *Monatsschrift Kinderheilkunde* 2012; **160**(4): 395-406.
